# Red yeast rice lowers cholesterol in physicians - a double blind, placebo controlled randomized trial

**DOI:** 10.1186/1472-6882-13-178

**Published:** 2013-07-18

**Authors:** Veronique Verhoeven, Maja Lopez Hartmann, Roy Remmen, Johan Wens, Sandra Apers, Paul Van Royen

**Affiliations:** 1 The academic center for primary and interdisciplinary care, Faculty of Medicine and Health Sciences, University of Antwerp, Antwerp, Belgium; 2 Natural products and food – Research & Analysis, Faculty of Pharmaceutical, Biomedical and Veterinary Sciences, Universiteitsplein 1, Wilrijk 2610, Belgium

**Keywords:** Cardiovascular prevention, Statins, Red yeast rice, Cholesterol

## Abstract

**Background:**

In recent years**,** red yeast rice (RYR) supplements have been marketed aggressively as a natural way to lower cholesterol; however, the large majority of commercially available products have not been studied according to current research standards.

**Methods:**

In a double blind placebo controlled randomized trial, 52 physicians and their spouses with a total cholesterol level of > 200 mg/dL were randomly allocated to receive a RYR extract or placebo for 8 weeks. As a primary outcome measure, we compared the before-after difference in lipid levels between both groups. As secondary outcome measures we looked at side-effects, CK elevation and a change in cardiovascular risk.

**Results:**

LDL (low density lipoprotein) cholesterol was lowered with 36 mg/dL (22%) and total cholesterol with 37 mg/dL (15%) in the intervention group. This result was statistically significant as compared to the control group, in which no reduction in total cholesterol and LDL was observed (p < 0.001). There was no marked difference in CK (creatine kinase)-elevation or reported side-effects between study groups. In 5/31 participants in the intervention group, the lipid lowering effect resulted in lower cardiovascular risk as measured with SCORE (Systematic COronary Risk Evaluation).

**Conclusions:**

The RYR formulation under study was effective in lowering cholesterol and LDL cholesterol in this study population. RYR therapy may be an attractive and relatively well studied alternative in patients who are intolerant for statins or who have objections against pharmacological lipid lowering. However, consumers need to be warned that the actual content of commercially available preparations is not assured by governmental regulations, which raises effectiveness and safety issues.

**Trial registration:**

Clinicaltrials.gov, nr: NCT01558050

## Background

In recent years, red yeast rice (RYR) supplements have encountered increasing research interest for their possible lipid-lowering effects. Whereas the evidence in favor of these products is still being developed and refined, dietary supplement industries have proceeded without delay to produce a number of RYR formulations. These food supplements are marketed and mediatized as a “natural” alternative to lipid lowering drugs and as a solution for patients who experience statin-associated myalgia.

RYR, which has been used for centuries in China as a food colorant and in traditional medicine “to invigorate the body, aid in digestion, and revitalize the blood” [1], is produced by culturing the yeast *Monascuspurpureus* on rice. This process results in the production of a mixture of monacolins, which are inhibitors of hydroxymethylglutaryl-coenzyme A (HMG-CoA) reductase and thus inhibit cholesterol synthesis in the liver. Monacolin K is chemically identical to the statin commercialized as lovastatin.

RYR products are now widely available as over-the-counter products; however, monacolin levels per gram of labeled “active product” are not standardized and show substantial variability among marketed products [2]. It is not possible to make a comparison between formulations, and information on labels and actual content may differ.

Since functional foods are becoming more and more popular, physicians should be prepared to answer questions and to determine their position regarding alternative therapies. However, as in many countries, the efficacy of none of the RYR preparations on the Belgian market has been studied in a satisfactory way, making any informed advice for consumers impossible. Therefore, with the help of physicians in the field who acted as study participants, we conducted a randomized, double-blind, placebo-controlled trial to determine the efficacy of red yeast rice as dietary supplement in reducing lipids.

## Methods

The study was conducted from March to June 2012. Participants were medical doctors and their partners who were invited via an advertisement in a free medical journal in Flanders, Belgium. Eligible candidates were men and women over age 18 with a total fasting cholesterol level above 200 mg/dL. Exclusion criteria were present treatment with statins, triglyceride values >400 mg/dL which makes LDL estimation inaccurate [3], changes in medication or food supplements which affect lipid levels during the study (antihypertensive medication, especially diuretics, ezetimibe, fibrates, omega-3 fatty acid food supplements). No diet was imposed during the study.

We used a commercially available product containing 1,5% Monacoline K and a placebo which looked exactly the same as the original product. Every capsule contained 5,025 mg of Monacolin K; furthermore the product contained Ubiquinone (Co-enzyme Q-10) 30 mg, Procyanidins (Vitisvinifera L) 20 mg and Lecithin 100 mg. The content of the capsules was analyzed by an independent laboratory, certified by the Belgian Federal Public Service for health, food chain safety and environment (laboratory ECCA nv, Belgium, report number 11–008161). Monacolin K was detected using an in-house liquid chromatography–mass spectrometry method. The formulation was tested for the presence of heavy metals (As, Cd, Hg, Pb), benzo-a-pyrene and mycotoxins (citrinin).

A sample size calculation based on SD (standard deviation) values in previous studies, determined that 40 patients were needed to establish a mean difference in LDL-cholesterol reduction of 15% between both groups (power 0.80, significance level 0.05). LDL reduction was chosen for sample size calculation because it is the most commonly used surrogate endpoint for cardiovascular disease reduction, and it is the primary target for statin therapy in cholesterol-lowering guidelines [4-7].

Participants were randomized by the chief investigator (VV) to the control or intervention group in an 1:1 ratio, by randomly generating even (for intervention) and uneven (for control) numbers using a computer program (http://www.random.org). No stratification for age, sex or cholesterol level was performed. If a couple of a doctor and his partner participated, a random number was produced for the doctor, and his/her partner was automatically allocated to the other group. The product and placebo were administered in a neutral, white container and labeled with a colored tag. Study participants were instructed to take 2 capsules every evening for 8 weeks.

Study groups were blinded to participants as well as researchers. After completion of the analyses, the key for control and intervention group (the color of the tag for placebo and for active product) was obtained from the manufacturer.

Blood samples were taken twice during the study period: a baseline measurement before starting with the dietary supplement and a second measurement after eight weeks. Fasting plasma cholesterol, HDL (high density lipoprotein), LDL and triglycerides were measured; the second measurement included CK levels.

At the end of the study, participants filled in a structured questionnaire regarding cardiovascular risk status and eventual side-effects. They were specifically asked for the following possible side-effects: muscle ache, muscle weakness, muscle stiffness, muscle cramps, arthralgia, sleeping disorders, depression, hair loss, and erectile dysfunction [8,9]. Furthermore, participants could report other possible side-effects.

As a primary outcome measure we compared the before-after intervention difference in lipid levels, especially LDL cholesterol, between the intervention and the control group, using the independent samples t-test. As secondary outcome measures we looked at side-effects, CK elevation and a change in cardiovascular risk.Statistical analyses were performed using SPSS, SPSS Inc, Chicago, Illinois, USA.

The study protocol got ethical approval by the university board of Antwerp University (number 01558050), and it was registered at clinicaltrials.gov, nr NCT01558050. Participants provided written consent for participation in the study.

## Results

Fifty-six participants were recruited for the study. Two study candidates were excluded because of triglyceride values > 400. Fifty-four participants were randomized by generating even and uneven numbers; the fact that more participants were allocated to the intervention group (31versus 23) was not deliberate. Two eligible candidates agreed to participate in the study and provided a first blood sample, but they failed to start to take the study product. They were excluded from analysis; thus a per protocol analysis was performed on 52 participants. Their baseline characteristics are shown in Table 1.

**Table 1:** Baseline characteristics of study participants

	**Intervention group (n = 31)**	**Placebo group (n = 21)**	p-value*
Age (mean, SD)	55 (7)	55 (11)	0,97
%Male /%Female	61/39	43/58	0,26
Mean total cholesterol in mg/dl (SD)	244 (28)	233 (26)	0,16
Mean HDL in mg/dL (SD)	66 (17)	60 (16)	0,21
Mean LDL in mg/dL (SD)	154 (29)	148 (22)	0,42
Mean TG in mg/dL (SD)	119 (53)	120 (62)	0,93
Mean systolic blood pressure (SD)	118 (8,5)	123 (10,8)	0,10
Mean diastolic blood pressure (SD)	74 (8,1)	77 (6,7)	0,14
Smokers	0	1	0,40
Former smokers	12	5	0,37
Diabetes/impaired glucose tolerance	1	2	0,56
Family history of cardiovascular risk**	2	2	1,00
Cardiovascular risk profile***, median risk (range)	2 (0–5)	2 (0–4)	0,21

*Paired t-test, chi^2^ test, Fischer’s exact test or Mann–Whitney U test.

**Defined as having a father or brother with an ischemic event (cardiac, cerebral, or peripheral) before the age of 55 and/or a mother or sister with an ischemic event before the age of 65.

***Ten-year risk of fatal CVD based on Belgian SCORE table (by gender, age, systolic blood pressure, total cholesterol and smoking status [6].

**Figure 1:**
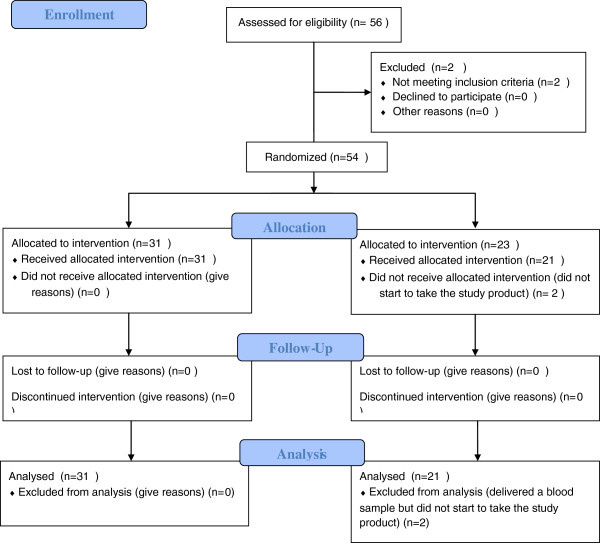
Consort 2010 flow diagram.

The flow of participants through the study is shown in Figure 1.

**Figure 2:**
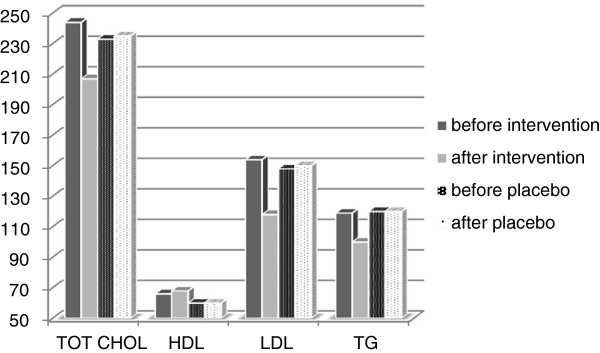
Absolute before and after levels of serum lipids in study participants.

Table 2 compares the absolute and relative difference in serum lipid levels before and after the intervention in both groups. Before- and after lipid profiles are shown in Figure 2.

**Table 2:** Mean absolute and relative difference in serum lipid levels (levels at beginning minus levels at the end)

**Mean difference in serum lipid level (mg/dL)**	**Intervention group**	**Placebo group**	**95% CI of difference (%)**	**p-value**
Diff TOTchol	37,06 mg/dL	-1,86	-52,01;-25,83	<0,001
(%)	(14,62)	(-1,16)	(-20,89; -10,68)	
Diff HDL	-1,90 mg/dL	0,43 (0,27)	-2,20;6,87	0,31
(%)	(-2,99)		(-3,46; 9,99)	
Diff LDL	35,87 mg/dL	-2,04 (-1,65)	-49,77;-26,05	<0,001
(%)	(22,17)		(-30,46; -17,17)	
Diff TG	19,13 mg/dL	-0,29 (-4,97)	-38,51; -0,32	0,05
(%)	(13,79)		(-32,68; -4,83)	

The difference in LDL was normally distributed (Kolmogorov Smirnov test = 0,53) but there was some visible skewness to the left. Practically this means that 20% of participants in the intervention group (6/31) experienced an effect on LDL of 0%-10%. The magnitude of the effect in the intervention group ranged from -8.03% to 40.46%, with a mean difference of 22.17% and a median difference of 25,47%.

Within de intervention group, the lipid lowering effect was not influenced by age or gender. There was a tendency towards a lower effect in participants with lower self-reported compliance (16,5% LDL reduction versus 23,8%, p = 0,17). Low compliance was defined as non-intake of the supplement for > =10% of the time.

The cardiovascular risk according to SCORE changed after the intervention in 5/31 subjects in the intervention group; in 3 participants the 10-year risk of fatal cardiovascular risk changed from 2 to 1%, in 2 participants from 4 to 3%. In the control group, the risk changed from 3 to 4% in 1/21 participant.

Mild CK elevation (less than twice the upper margin of the normal CK reference level) was observed in 3/21 control subjects and in 5/31 participants in the intervention group. In one patient in the intervention group these CK-elevation was accompanied by muscle ache.

Self-reported possible side-effects were as follows: muscle ache 6 times (4 in intervention group and 2 in control group), muscle weakness 1 (0/1), muscle stiffness 2 (2/0), muscle cramps 4 (3/1), insomnia 2 (1/1), erectile dysfunction 1 (0/1), belches 1 (1/0), pruritus 1 (0/1), liver pains 1 (0/1). No participants discontinued the intake of the study product; however one person in the intervention group stated that, although he did not discontinue the product for the study, the side-effects (muscle ache) were unacceptable to him.

## Discussion

This study demonstrated that consumption of the red rice extract under study resulted in a statistically significant lowering of cholesterol levels. The LDL concentration, the primary target, was reduced with 22% in the intervention group. A marked variability in the magnitude of the response was observed. This is similar to the inter-individual variation in response to statins, resulting in “poor” and “good” responders, in which genetic factors, which influence statin metabolism, seem to play an important role [10].

The sample size of this study was sufficient to assess effectiveness of the product under study, but does not allow to adequately assess side-effects. Although the difference was not statistically significant, there appeared to be slightly more muscle complaints in the intervention group. Another limitation of this trial is the short follow up time, and the fact that no baseline CK-level was determined.

The formulation under study contained 5,025 mg of Monacolin K per capsule, resulting in a daily dose of 10,05 mg, which is a higher dose than most commercially available products. Other monacolins, which are the result of the fermentation process, were not quantified in this study. However, they also inhibit hydroxymethylglutaryl-coenzyme A (HMG-CoA) reductase and thus can contribute to the observed lipid lowering effect. In future research it would be useful to do a more thorough characterization of the different fermentation products, in order to be able to determine the effect of each of them. Furthermore, the possibility of spiking cannot be excluded for this product. In the absence of valid estimations of a “natural” ratio of Monacolin K / other monacolins, it is presently impossible to determine whether all Monacolin K is of natural origin in this formulation. Next to monacolins, the study product contains other “active” substances: lecithin and procyanidins are, without proof of a clinical effect, believed to influence cholesterol metabolism. Furthermore the manufacturer added coenzyme Q10 (ubiquinone) aiming to reduce the risk of side-effects. Coenzyme Q10 deficiency has been proposed to play a role in 3-hydroxy-3-methyl-glutaryl coenzyme A reductase inhibitor (statin)-induced myopathies; evidence on the clinical benefit of coenzyme Q10 supplementation in (presumed) statin-induced muscle complaints is equivocal [11,12] and a possible effect may depend on genetic factors [13]. The perceived effect in this study is the result of the formulation as a whole, but it is unknown whether the combination of the different substances in the study product has had an additive, synergistic or even antagonistic effect on cholesterol levels.

A growing number of studies evaluated effectiveness and safety of RYR in lowering of plasma lipids. There is also some evidence regarding effectiveness in secondary prevention from a large multicenter, randomized, double blind placebo-controlled study in 4870 Chinese patients with a history of myocardial infection and moderately hypercholesterolemia. This study showed, over a period of 4,5 years, a reduction by one third in cardiovascular deaths and in the need for coronary revascularization. In high risk subgroups (diabetes, hypertension) a reduction in coronary events was shown [14,15].

Whether RYR preparations should be considered as dietary supplements or as drugs is still under debate. In the US, the Food and Drug Administration (FDA) considers these supplements as (unapproved) drugs when they contain a specific, standardized amount of lovastatin [2]. In Europe this restriction has not yet been imposed. In addition to monacolins, most commercially available products contain other active substances such as Coenzym Q10, isoflavones, probiotics and others, and manufacturers commonly do not disclose levels of active substances in their preparations. In some products, potentially harmful levels of suspected nephrotoxins have been found [2]. Evidently, the lack of dietary supplement regulations raises possible safety concerns.

Which are possible indications for RYR therapy in clinical practice? Firstly, RYR may be an alternative for patients with a history of statin-related adverse effects [16]. Myopathy occurs in approximately 10% of statin-treated patients, which a spectrum ranging from rhabdomyolysis to (in a vast majority) myalgias without elevation in serum creatine kinase (CK) levels. Although a link with statin use can often not be established with certainty, true as well as perceived statin intolerance can undermine compliance [9,17]. In patients intolerant to synthetic statins, RYR has shown to be an alternative which is moderately efficient, acceptable and supported well [18,19]. However, it is no surprise that myopathy, including rhabdomyolysis, has also been reported in patients taking products containing RYR [20,21]. It should be kept in mind that RYR, as any other HMG-CoA reductase inhibitor, can cause myopathy, although up to now it does not seem to be a common finding.

A second group of potential users are people who refuse to take statins for philosophical reasons [22]. The growing group of patients who have an aversion to chronic drug intake will certainly lead to more questions in the doctor’s office for alternative therapies [23]. Functional foods like RYR can be an attractive and relatively well studied option for these patients. Evidently they need to be informed that also naturally occurring statins contain pharmacologically active molecules which represent a (low) possibility of side-effects [23].

A third, more speculative use of RYR may in be people with elevated cholesterol/ LDL levels, but who still have a low cardiovascular risk when other risk factors are taken into account. There is compelling evidence for statin therapy for secondary prevention but for primary prevention, cost-effectiveness and effect on quality of life are less clear [24-26]. Primary prevention, even in low risk individuals, has both strong proponents and strong opponents. Whereas conclusive evidence is lacking, annual spending on statins represents an enormous expenditure for the health care budget [27]. In ten years’ time, the amount of statin DDDs (defined daily doses) delivered was multiplied by 20 in Belgium, representing a cost of 225607858 euros, which is 7% of the overall expenditure for ambulatory drugs in Belgium [28]; atorvastatin and rosuvastatin rank respectively first and third in the list of net drug expenses in the ambulatory sector [29]. At the same time, although not (yet) supported by direct evidence from trials [30,31] target values for LDL cholesterol keep being set lower [32]. In the absence of definitive evidence and to relieve the health care budget, people at low risk who still wish to lower their lipid levels may use functional foods like RYR at their own expense. However, an alternative would be to use a statin at peoples’ own expense - evidently the government has an crucial responsibility in determining the conditions for reimbursement of statins.

## Conclusion

In this study, a commercially available RYR product showed to be efficient in lowering cholesterol values, with a mean LDL difference of 22%. Possible indications for RYR therapy are intolerance of statins or refusal to take synthetic drugs in people in whom lipid lowering therapy is indicated. It has to be kept in mind that also naturally occurring statins in RYR represent a risk of muscular and other side-effects, although the risk appears to be low. Furthermore, the lack of regulation in the food supplement industry raises issues concerning efficiency and safety. Studies with longer follow up are warranted to asses long-term safety and effectiveness.

Red rice in general should not be promoted as “a natural way to lower your cholesterol [33]”, but physicians should advice their patients on specific products that contain proper formulations and that have been studied according to current research standards. If these precautions are taken into account, these products can be an attractive alternative to traditional statins.

## Competing interests

Decola Nutraceutics Belgium gave us permission to use their commercially available product Lipeq-10 for our study. Furthermore, they provided the study product and assisted with production of the placebo. Decola had no further financial, methodological or any other input in design, execution and reporting of this study. Decola had no access to the study protocol, names of participating physicians or raw study data. Before starting the study, it was agreed specifically that the study would be published, regardless of the results.

## Authors’ contributions

VV initiated the study, was the primary researcher and wrote the initial draft. MLH, RR and JW participated in study design, data collection and writing the manuscript. PVR had the general supervision, helped funding the study with departmental resources, and commented on the manuscript. SA assisted with methodological issues and with interpretation of the characterization of the study product. All authors read and approved the final manuscript.

## Pre-publication history

The pre-publication history for this paper can be accessed here:

http://www.biomedcentral.com/1472-6882/13/178/prepub

## References

[B1] Ying Sing S T’ien Jung K'ai W Chinese technology in the seventeenth century Sun E-T and Sun S-C, transl 1966 London: Pennsylvania State University Press 291 294

[B2] Gordon RY Cooperman T Obermeyer W Becker DJ Marked variability of monacolin levels in commercial red yeast rice products: buyer beware! Arch Intern Med 2010 170 1722 1727 10.1001/archinternmed.2010.382 20975018

[B3] Rifai N Warnick GR McNamara JR Belcher JD Grinstead GF Frantz ID Jr Measurement of low-density-lipoprotein cholesterol in serum: a status report Clin Chem 1992 38 150 160 1733589

[B4] Law MR Wald NJ Rudnicka AR Quantifying effect of statins on low density lipoprotein cholesterol, ischaemic heart disease, and stroke: systematic review and meta-analysis BMJ 2003 326 1407 1408 10.1136/bmj.326.7404.1407 12829554 PMC162260

[B5] Mihaylova B Emberson J Blackwell L Keech A Simes J Barnes EH Voysey M Gray A Collins R Baigent C Cholesterol Treatment Trialists’ (CTT) Collaborators The effects of lowering LDL cholesterol with statin therapy in people at low risk of vascular disease: meta-analysis of individual data from 27 randomised trials Lancet 2012 380 581 590 22607822 10.1016/S0140-6736(12)60367-5 PMC3437972

[B6] Bulbulia R Bowman L Wallendszus K Parish S Armitage J Peto R Collins R Heart Protection Study Collaborative Group Effects on 11-year mortality and morbidity of lowering LDL cholesterol with simvastatin for about 5 years in 20,536 high-risk individuals: a randomised controlled trial Lancet 2011 378 2013 2020 22115874 10.1016/S0140-6736(11)61125-2 PMC3242163

[B7] The Fifth Joint Task Force of the European Society of Cardiology and Other Societies on Cardiovascular Disease Prevention in Clinical Practice (constituted by representatives of nine societies and by invited experts) European guidelines on cardiovascular disease prevention in clinical practice (version 2012) Eur Heart J 2012 33 1635 1701 22555213 10.1093/eurheartj/ehs092

[B8] Harper CR Jacobson TA Evidence-based management of statin myopathy Curr Atheroscler Rep 2010 12 322 30 10.1007/s11883-010-0120-9 20628837

[B9] Mancini GB Baker S Bergeron J Fitchett D Frohlich J Genest J Gupta M Hegele RA Ng D Pope J Diagnosis, prevention, and management of statin adverse effects and intolerance: proceedings of a Canadian Working Group Consensus Conference Can J Cardiol 2011 27 635 662 10.1016/j.cjca.2011.05.007 21963058

[B10] Sirtori CR Mombelli G Triolo M Laaksonen R Clinical response to statins: mechanism(s) of variable activity and adverse effects Ann Med 2012 44 419 432 10.3109/07853890.2011.582135 21623698

[B11] Caso G Kelly P McNurlan MA Lawson WE Effect of coenzyme Q10 on myopathic symptoms in patients treated with statins Am J Cardiol 2007 99 1409 1412 10.1016/j.amjcard.2006.12.063 17493470

[B12] Bookstaver DA Burkhalter NA Hatzigeorgiou C Effect of coenzyme Q10 supplementation on statin-induced myalgias Am J Cardiol 2012 110 526 529 10.1016/j.amjcard.2012.04.026 22608359

[B13] Marcoff L Thompson PD The role of coenzyme Q10 in statin-associated myopathy - a systematic review J Am Coll Cardiol 2007 49 2231 2237 10.1016/j.jacc.2007.02.049 17560286

[B14] Lu Z Kou W Du B Wu Y Zhao S Brusco OA Morgan JM Capuzzi DM Li S Chinese Coronary Secondary Prevention Study Group Effect of Xuezhikang, an extract from red yeast Chinese rice, on coronary events in a Chinese population with previous myocardial infarction Am J Cardiol 2008 101 1689 1693 10.1016/j.amjcard.2008.02.056 18549841

[B15] Li JJ Lu ZL Kou WR Chen Z Wu YF Yu XH Zhao YC Chinese Coronary Secondary Prevention Study Group. Beneficial impact of Xuezhikang on cardiovascular events and mortality in elderly hypertensive patients with previous myocardial infarction from the China Coronary Secondary Prevention Study (CCSPS) J Clin Pharmacol 2009 49 947 956 10.1177/0091270009337509 19602720

[B16] Reinhart KM Woods JA Strategies to preserve the use of statins in patients with previous muscular adverse effects Am J Health Syst Pharm 2012 69 291 300 10.2146/ajhp100700 22302254

[B17] Eckel RH Approach to the patient who is intolerant of statin therapy J Clin Endocrinol Metab 2010 95 2015 2022 10.1210/jc.2009-2689 20444930

[B18] Venero CV Venero JV Wortham DC Thompson PD Lipid-lowering efficacy of red yeast rice in a population intolerant to statins Am J Cardiol 2010 105 664 666 10.1016/j.amjcard.2009.10.045 20185013

[B19] Becker DJ Gordon RY Halbert SC French B Morris PB Rader DJ Red yeast rice for dyslipidemia in statin-intolerant patients: a randomized trial Ann Intern Med 2009 150 830 839 W147-9 10.7326/0003-4819-150-12-200906160-00006 19528562

[B20] Lapi F Gallo E Bernasconi S Vietri M Menniti-Ippolito F Raschetti R Gori L Firenzuoli F Mugelli A Vannacci A Myopathies associated with red yeast rice and liquorice: spontaneous reports from the Italian Surveillance System of Natural Health Products Br J ClinPharmacol 2008 66 572 574 10.1111/j.1365-2125.2008.03224.x PMC2561108 18637891

[B21] Mueller PS Symptomatic myopathy due to red yeast rice Ann Intern Med 2006 145 474 475 10.7326/0003-4819-145-6-200609190-00021 16983142

[B22] Gordon RY Becker DJ The role of red yeast rice for the physician Curr Atheroscler Rep 2011 13 73 80 10.1007/s11883-010-0145-0 21061097

[B23] Sirtori CR Galli C Anderson JW Sirtori E Arnoldi A Functional foods for dyslipidaemia and cardiovascular risk prevention Nutr Res Rev 2009 22 244 261 20003590 10.1017/S0954422409990187

[B24] Taylor F Ward K Moore TH Burke M Davey Smith G Casas JP Ebrahim S Statins for the primary prevention of cardiovascular disease Cochrane Database Syst Rev 2011 1 CD004816 21249663 10.1002/14651858.CD004816.pub4 PMC4164175

[B25] Prasad V Vandross A Cardiovascular primary prevention: how high should we set the bar? Arch Intern Med 2012 172 656 659 10.1001/archinternmed.2012.812 22529231

[B26] Tonelli M Lloyd A Clement F Conly J Husereau D Hemmelgarn B Klarenbach S McAlister FA Wiebe N Manns B Alberta Kidney Disease Network Efficacy of statins for primary prevention in people at low cardiovascular risk: a meta-analysis CMAJ 2011 183 E1189 E1202 21989464 10.1503/cmaj.101280 PMC3216447

[B27] Stagnitti M Trends in statins utilization and expenditures for the U.S. civilian noninstitutionalized population, 2000 and 2005 MEPS Statistical Brief. No. 205 http://meps.ahrq.gov/mepsweb/data_files/publications/st205/stat205.pdf. Accessed September 11, 2012

[B28] Roberfroid D Dubois C Vrijens F Camberlin C Farfan-Portet M Statines in Belgie: evolutie in het gebruik en invloed van het terugbetalingsbeleid. KCE report, 2010, English versionhttps://kce.fgov.be/nl/publication/report/statines-in-belgie-evolutie-in-het-gebruik-en-invloed-van-het-terugbetalingsbelei accessed 11/9/2012

[B29] http://www.riziv.fgov.be/drug/nl/statistics-scientific-information/pharmanet/info-spot/2011-09-22/index.htm, accessed 26/9/2012

[B30] Krumholz HM Hayward RA Shifting views on lipid lowering therapy BMJ 2010 341 c3531 10.1136/bmj.c3531 20667950

[B31] Lebenthal Y Horvath A Dziechciarz P Szajewska H Shamir R Are treatment targets for hypercholesterolemia evidence based? Systematic review and meta-analysis of randomised controlled trials Arch Dis Child 2010 95 673 680 10.1136/adc.2008.157024 20515970

[B32] Martin SS Blumenthal RS Miller M LDL cholesterol: the lower the better Med Clin North Am 2012 96 13 26 10.1016/j.mcna.2012.01.009 22391248

[B33] Campbell A Natural ways to lower your cholesterol Diabetes Self Manag 2010 27 40 46 20575327

